# Association of Fetal Lung Development Disorders with Adult Diseases: A Comprehensive Review

**DOI:** 10.3390/jpm14040368

**Published:** 2024-03-29

**Authors:** Alexey V. Yaremenko, Nadezhda A. Pechnikova, Konstantinos Porpodis, Savvas Damdoumis, Amalia Aggeli, Papamitsou Theodora, Kalliopi Domvri

**Affiliations:** 1 Brigham and Women’s Hospital, Harvard Medical School, Boston, MA 02115, USA; 2 Oncology Unit, Pulmonary Department, George Papanikolaou Hospital, School of Medicine, Aristotle University of Thessaloniki, 54636 Thessaloniki, Greece; kporpodis@yahoo.gr (K.P.); dcsavvas@auth.gr (S.D.); 3 Laboratory of Chemical Engineering A’, School of Chemical Engineering, Faculty of Engineering, Aristotle University of Thessaloniki, 54636 Thessaloniki, Greece; nikanobelevka@gmail.com (N.A.P.); aggeli@auth.gr (A.A.); 4 Saint Petersburg Pasteur Institute, Saint Petersburg 197101, Russia; 5 Laboratory of Histology-Embryology, School of Medicine, Aristotle University of Thessaloniki, 54636 Thessaloniki, Greece; thpapami@auth.gr; 6 Pathology Department, George Papanikolaou Hospital, Aristotle University of Thessaloniki, 54636 Thessaloniki, Greece

**Keywords:** lung development, neonatal outcomes, fetal respiratory diseases, epigenetic modifications, transgenerational effects, adult respiratory diseases, preventive interventions

## Abstract

Fetal lung development is a crucial and complex process that lays the groundwork for postnatal respiratory health. However, disruptions in this delicate developmental journey can lead to fetal lung development disorders, impacting neonatal outcomes and potentially influencing health outcomes well into adulthood. Recent research has shed light on the intriguing association between fetal lung development disorders and the development of adult diseases. Understanding these links can provide valuable insights into the developmental origins of health and disease, paving the way for targeted preventive measures and clinical interventions. This review article aims to comprehensively explore the association of fetal lung development disorders with adult diseases. We delve into the stages of fetal lung development, examining key factors influencing fetal lung maturation. Subsequently, we investigate specific fetal lung development disorders, such as respiratory distress syndrome (RDS), bronchopulmonary dysplasia (BPD), congenital diaphragmatic hernia (CDH), and other abnormalities. Furthermore, we explore the potential mechanisms underlying these associations, considering the role of epigenetic modifications, transgenerational effects, and intrauterine environmental factors. Additionally, we examine the epidemiological evidence and clinical findings linking fetal lung development disorders to adult respiratory diseases, including asthma, chronic obstructive pulmonary disease (COPD), and other respiratory ailments. This review provides valuable insights for healthcare professionals and researchers, guiding future investigations and shaping strategies for preventive interventions and long-term care.

## 1. Introduction

Fetal lung development is a complex and precise biological process, crucial for establishing the lungs, a vital organ in the human body. This intricate process involves a variety of cells, proteins, and genes, such as progenitor cell states, neuroendocrine cell subtypes, fibroblast growth factor 10, *FoxP1*, *FoxP1*, *THRB*, *EGR3*, *ETV1*, and others. It also utilizes diverse developmental mechanisms, including IL-33/ILC2/IL-13, NOTCH, TGFB, and sonic hedgehog signaling, among others [1,2,3,4,5,6,7,8,9,10,11]. These elements are fundamental in forming structures essential for respiration, managing the critical exchange of oxygen and carbon dioxide, vital for cellular sustenance and function [12]. 

Nevertheless, this labyrinthine process of lung development can be subjected to disruptions because of different reasons (premature births, complications during pregnancy, undernutrition, infections, etc.) [13,14,15,16], leading to a wide spectrum of disorders affecting lungs not only in childhood but also in adult age [17]. Traditionally, these disorders have been scrutinized primarily for their immediate implications on neonatal health. Yet, in an intriguing shift, contemporary research is increasingly probing their potential correlation with adult diseases, providing a more longitudinal perspective on health outcomes [13,18].

To delve deeper into the nuances of these disorders and their potential ramifications, it is pivotal to first chart the sequential milestones of fetal lung maturation. Fetal lung development unfolds through five integral stages: embryonic, pseudoglandular, canalicular, saccular, and alveolar [19]. Each stage is characterized by a unique sequence of morphological transformations, progressively sculpting a fully functional respiratory organ [2,19,20]. Disruptions at any stage can lead to conditions like con-genital diaphragmatic hernia or pulmonary hypoplasia, which, while immediately impactful on neonatal health, may also have long-term effects and predispose individuals to chronic respiratory ailments extending into adulthood [21,22,23,24,25,26,27,28].

Thus, in many cases, fetal lung malformations are directly linked to lung diseases such as COPD, asthma, and interstitial lung disease in adults [27,29,30], which, along with other pulmonary diseases, contribute significantly to global morbidity and mortality. These conditions, coupled with other lung-related diseases, underscore the critical need to investigate the relationships between such ailments and their multifaceted origins, including fetal lung abnormalities [31,32]. Hence, gaining a deeper understanding of these relationships has the potential to provide pivotal insights, propelling forward advancements in early detection, tailored treatments, and innovative preventive measures.

In this review, we aim to shed light on the vast body of knowledge surrounding the relationship between fetal lung development disorders and their long-term impact on adult health. We examine the complex biological, genetic, and environmental factors that affect lifelong health trajectories. Our in-depth analysis highlights the intersections of research, clinical practice, and patient care. Our discourse revolves around the burgeoning recognition that early life experiences profoundly shape adult health outcomes. This concept, steadily gaining traction, is revolutionizing the contours of contemporary medicine and public health strategies.

## 2. Fetal Lung Development: An Overview

**Figure 1:**
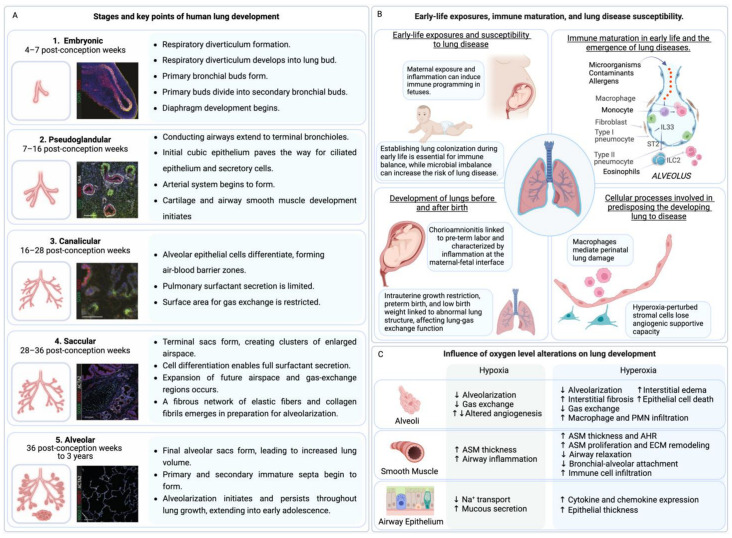
**Fetal lung development: stages, influential factors, and its pivotal role in postnatal health.** (**A**) Stages and key points of human lung development: The different stages and important aspects of human lung development are shown in diagrams. These stages include embryonic, pseudoglandular, canalicular, saccular, and alveolar. During the embryonic stage, primary branches and SOX2 (Transcription Factor SOX-2)/SOX9 (Transcription Factor SOX-9) co-expression in the tips are visible. Ongoing tip SOX2/SOX9 co-expression and airway differentiation are depicted in the pseudoglandular stage. The canalicular stage shows SOX9^+^/SOX2^−^ distal tips, and alveolar differentiation begins. In the saccular stage, distal tips disappear and alveolar differentiation progresses, with the expression of SOX9 (cartilage), SOX2 (airway cells), and ACTA2 (Actin Alpha 2, Smooth Muscle). The postnatal alveolar stage shows continued growth and septal formation, with the expression of NKX2-1 (NK2 Homeobox 1) (lung epithelium), FOXF1 (Forkhead Box F1) (mesenchyme), and ACTA2. The SOX9+ distal tips are no longer visible at this stage. Cryosections are reproduced from [19]. Text descriptions are adapted from [33]. (**B**) Early-life exposures can affect respiratory disease development. Maternal factors like diet, smoking, and medication use can impact fetal immune programming. Early-life lung colonization is crucial for immune response shaping. Changes in lung development and exposure can lead to respiratory diseases later in life. Abnormal lung structure from preterm birth or chorioamnionitis-associated bronchopulmonary dysplasia can increase susceptibility to respiratory complications [30]. IL: interleukin; ST: suppression of tumorigenicity; ILC: innate lymphoid cell; Th: T-helper; PH: pulmonary hypertension. (**C**) Influence of oxygen level alterations on lung development: Both low and high oxygen levels can lead to vascular dysmorphogenesis, which increases the risk of wheezing and asthma in children [34].

Fetal lung development is a critical and multifaceted process that sets the foundation for respiratory health after birth. This complex developmental journey holds the key to understanding the potential impact of disruptions on both immediate neonatal outcomes and long-term health. By unravelling the mysteries of this progression, healthcare professionals and researchers can uncover essential insights, enabling preventive measures and interventions that safeguard respiratory health from life’s inception (Figure 1).

### 2.1. Stages of Fetal Lung Development

Fetal lung development is a dynamic process, highly coordinated and regulated, that unfolds through five essential stages (Figure 1A) [19], each contributing to the formation of fully functional lungs capable of sustaining postnatal respiration.

During the embryonic stage, which spans approximately from week 4 to 7 of gestation (or 3–6), the primitive lung bud, or respiratory diverticulum, arises from the foregut endoderm [19,35,36,37]. This bud further bifurcates into the primary bronchi and pulmonary buds, initiating the branching morphogenesis that will give rise to the entire conducting airway tree [37]. In addition, the development of the diaphragm is observed at this stage, which is related to the lateral folding of the body wall enclosing the foregut endoderm, and the subsequent growth of the septum transversum, a block of connective tissue between the heart and future liver. This growth process contributes to the separation of the thoracic and abdominal cavities, marking a crucial stage in formation of the diaphragm [37]. At the culmination of the embryonic stage, the foundational structures such as the larynx, trachea, initial stages of the lungs, distinct lung lobes, and intricate bronchopulmonary segments have intricately taken shape [37].

During the pseudoglandular stage, approximately week 7 to 16, pulmonary buds branch out extensively to form terminal bronchioles and primitive conducting airways, which now include an initial cubic epithelium transitioning to ciliated and secretory cells. Though this period lacks gas exchange structures, it introduces the pulmonary vascular system alongside the arterial system’s formation. Concurrently, cartilage and airway smooth muscle begin to develop. These changes lay down the essential architecture for the respiratory system, absent gas exchange capabilities, preparing for future lung maturation [35].

Further, from weeks 16 to 28, during the canalicular stage, the developing airways continue to branch out, forming the primitive respiratory bronchioles and thin-walled saccules, lined with capillaries. Despite the foundational work for gas exchange being established during this stage, the lungs remain premature and insufficiently developed to support independent life if birth were to occur [19,36,37].

Subsequently, the saccular stage extends from approximately week 28 to 36 [19,36,37]. This phase is marked by the emergence of mature alveoli with thin walls, ready to facilitate gas exchange. Capillaries proliferate, wrapping around the nascent alveoli, setting the stage for oxygen and carbon dioxide exchange. In parallel, the crucial surfactant production begins, reducing surface tension and preventing the alveoli from collapsing during exhalation [19,36,37].

Lastly, the alveolar stage, which commences from around week 36 and extends into early childhood, sees the number and size of alveoli dramatically increase, contributing to a more intricate lung structure. Further, postnatal lung development continues, with alveoli proliferating and enlarging throughout childhood [19,36,37].

Therefore, the process of fetal lung maturation is complex and pivotal for a newborn’s ability to breathe independently and maintain good health in adulthood. From the embryonic stage through postnatal life, numerous factors contribute to this intricate process. However, there are still many unanswered questions about respiratory specification and branching morphogenesis, alveolar cell fate specification and maturation, and the diversity and profiling of human cells in the developing human lung. These questions include the role of Sonic Hedgehog (SHH) and Fibroblast Growth Factor (FGF) signaling in NK2 Homeobox 1 or Thyroid transcription factor-1 (NKX2.1^+^) lung progenitor cell specification, the molecular mechanisms of FGF and Wingless-Type MMTV Integration Site Family Member (WNT) in branching morphogenesis, and the exact signaling required for respiratory cell specification versus organization. Additionally, the timing of alveolar cell specification, signaling pathways regulating Alveolar Type I Cell (ATI) vs. Alveolar Type II Cell (ATII) cell fate choice, the role of glucocorticoid signaling in alveolar cell fate specification/maturation, and the significance of mesenchyme-epithelial cross-talk in this process are still unknown [8,19,35,38,39]. Understanding these stages and related unknowns is crucial, particularly in managing preterm births, where incomplete airway and lung development poses a significant threat. This knowledge is also vital for treating lung diseases like neonatal respiratory distress syndrome, emphasizing the need for comprehensive understanding to guide effective interventions. It guides clinicians in making informed decisions about interventions, such as the timing of corticosteroid administration to enhance lung maturity in preterm labor [40,41]. Deeply understanding these processes can also help prevent lung diseases in adulthood.

### 2.2. Key Factors Influencing Fetal Lung Maturation

Fetal lung maturation is a complex process that determines a newborn’s ability to breathe independently after birth. This process is influenced by various factors such as genetics, hormones, maternal health and lifestyle, and nutrition (Figure 1B) [30,42]. It is important to have a comprehensive understanding of these factors to manage preterm births and treat neonatal respiratory distress syndrome. Moreover, intrauterine developmental delay and low birth weight (LBW) are identified as risk factors for neonatal chronic lung disease. Kuiper-Makris et al. explored the link between LBW and lung function reduction and examined angiogenesis in rat models of intrauterine growth restriction on a low-protein diet. Their findings indicate a potential correlation between LBW and lower FEV1 and FVC values. Early life stages showed decreased angiogenic signaling and endothelial markers, with subsequent postnatal periods marked by reduced pulmonary capillaries and unchanged endothelial expressions but increased anti-angiogenic activity and proteolytic actions, leading to diminished lung elasticity. This suggests that LBW might contribute to impaired lung function into adulthood, highlighting the crucial phases of lung development affected by intrauterine growth conditions [43]. In addition, intrauterine developmental delay (IUCD) and meconium aspiration syndrome may increase the risk of pulmonary hypertension in newborns [44]. 

Also, a number of cohort studies indicate that both low and excessive fetal growth can have adverse effects on the body’s condition, leading to the development of various respiratory diseases. Measurements and main results of Dekker et al. reinforce this, showing that higher weights during the second and third trimesters, at birth, and at 12 months are linked to better lung function, as indicated by higher FEV1 and FVC. Conversely, greater weight at 3 months was associated with poorer lung function, evident from lower FEV1/FVC ratios and reduced forced expiratory flow at 75% of pulmonary volume. Restricted fetal weight growth correlated with lower lung function in childhood, which was partially dependent on infant weight growth patterns. Moreover, it was demonstrated accelerated fetal weight growth led to higher FVC and lower FEV1/FVC ratios only when accompanied by accelerated infant weight growth, highlighting the nuanced relationship between early growth patterns and lung health [45].

One of the main factors in lung development is genetics, which is a complex area that remains poorly understood. Genetic alterations can cause severe and often fatal pulmonary anomalies, which include those in the sonic hedgehog [10], fibroblast growth factor (Fgf-7, Fgf-10) [46,47], and thyroid transcription factor-1 (TTF-1/Nkx2. 1/TITF1) pathways, as well as ion transport [11,48]. Genetic mutations in surfactant protein B (SFTPB), surfactant protein C (SFTPC), ATP-binding cassette sub-family A member 3 (ABCA3), surfactant protein A1 (SFTPA1), and surfactant protein A2 (SFTPA2) can lead to critical respiratory distress syndromes in neonates or interstitial pulmonary pathologies during early childhood [49,50,51]. Congenital anomalies that affect the surfactant system, which is essential for regulating alveolar surface tension, can also cause respiratory distress in neonates. These anomalies include inherited deficits in surfactant protein B, variances within surfactant protein C, and anomalies associated with the ABCA3 transporter [51]. Disruptions in key developmental pathways related to genetics can lead to congenital lung malformations, highlighting the genetic complexity and sensitivity of lung development [52].

In addition to genetics, hormonal signaling plays a crucial role in lung development. Hormones such as sex-associated, glucocorticoid, thyroid, insulin-like growth factor-1 (IGF-1), and growth hormone-releasing hormones are involved in critical processes such as cell proliferation, differentiation, and surfactant production [53,54,55]. Glucocorticoids are crucial in the development and function of the lungs. For instance, glucocorticoids help in the maturation of type II pneumocytes, which are responsible for surfactant production, a substance essential for reducing surface tension in the alveoli and preventing lung collapse. So, in type II pneumocytes, glucocorticoids upregulate the expression of genes that code for surfactant proteins (SP-A, SP-B, SP-C, and SP-D) and enzymes involved in the synthesis of phospholipids, the major components of surfactant. This process ensures that the lungs are capable of efficient gas exchange by enhancing the production and secretion of surfactant, thereby facilitating lung expansion and function at birth [56,57,58,59]. 

Furthermore, corticosteroid signaling impacts the function of several proteins essential for alveolar fluid clearance; targets include the α-subunit of the epithelial sodium channel (αENaC) and the α1 and β1 subunits of the adenosine triphosphate (ATP)-dependent basolateral Na+/K+ pump, which are expressed in the respiratory epithelium and are crucial for sodium transport in the airways of humans [60]. Additionally, glucocorticoid receptor signaling plays a pivotal role in lung development by enhancing the differentiation of a newly identified proliferative mesenchymal progenitor cell (PMP) into matrix fibroblasts (MFBs). This process occurs partly through the direct activation of extracellular matrix-associated target genes such as Fn1, Col16a4, and Eln, as well as by influencing VEGF, JAK-STAT, and WNT signaling pathways. The absence of mesenchymal GR signaling hinders the differentiation of fibroblast progenitors into mature MFBs, leading to increased proliferation of SOX9+ alveolar epithelial progenitor cells and impeding the differentiation into mature alveolar type II (AT2) and AT1 cells. This intricate network of glucocorticoid receptor-mediated control over genes essential for mesenchymal progenitor differentiation into matrix fibroblasts subsequently regulates signaling mechanisms that govern the proliferation and differentiation of AT2/AT1 progenitor cells, underscoring the complex interplay by which glucocorticoid signaling facilitates fetal lung maturation [56].

Moreover, corticosteroids significantly impact lung development and maturation, particularly through their influence on antioxidant enzymes. These enzymes play a crucial role in protecting the developing lung tissue from oxidative stress, which can cause cellular damage and contribute to various pulmonary diseases (for example RDS, CDH, BPD). Corticosteroids induce the expression and activity of several key antioxidant enzymes, including superoxide dismutase, catalase, and glutathione peroxidase. The enhancement of these antioxidant defenses by corticosteroids is particularly important in the context of fetal and neonatal lung development, where the tissue is highly susceptible to oxidative stress. The increased expression and activity of these enzymes can help to mitigate the effects of reactive oxygen species, thereby protecting the developing pulmonary structure and function. This antioxidative action of corticosteroids is one of the mechanisms by which they contribute to the prevention of RDS in preterm infants and support the overall maturation of the lung [61,62].

In addition to genetics, hormonal signaling plays a crucial role in lung development. Hormones such as sex hormones, glucocorticoids, thyroid hormones, insulin-like growth factor-1 (IGF-1), and growth hormone-releasing hormone are involved in crucial processes such as cell proliferation, differentiation, and surfactant production [53,54,55]. For example, glucocorticoids promote the maturation of type II pneumocytes, which are responsible for surfactant production, thereby affecting lung function [56,57,58], and thyroid hormones regulate early lung morphogenesis and late maturation [63]. 

The effects of hormones on lung development are evident at the earliest stages of organismal development and have cellular differences. For example, it has been shown that the expression of glucocorticoid receptor (GR)α, thyroid hormone receptor (TR), retinoic acid receptor (RAR), and retinoid X receptor (RXR) in epithelial cells is detected at 13.5 weeks of gestation [64]. In another study, Habermehl et al. in vivo showed that glucocorticoid signaling in lung mesenchyme has a major effect on extracellular matrix (ECM) composition and activation of alveolar myofibroblasts [57]. These data reveal important new aspects of glucocorticoid action during lung development and may warrant careful analysis during corticosteroid therapy in preterm infants [57].

Also, lung development can be affected by the environment, which means not only the external environment, and external factors, but also the internal environment (oxygen, infections, etc.). For instance, maternal smoking, alcohol intake, and environmental toxin exposure have been linked with impaired fetal lung growth and functionality [65,66,67]. Likewise, optimal maternal nutrition can play a crucial role. Previous research indicates that maternal vitamin A deficiency may result in defective lung development and altered lung function in offspring [68,69,70,71]. Proper fetal circulation and oxygenation are also crucial for normal lung growth, making optimal blood flow and oxygen supply prerequisites for healthy lung development. Any disruptions in blood flow or oxygen supply can cause lung diseases such as bronchopulmonary dysplasia or disrupt the development of innate immunity (Figure 1C) [72,73,74,75]. Additionally, it is worth noting that lung development in female and male fetuses have different features, such as specific airway resistance being lower in females than in males, and more pronounced maturity of phospholipid profile in females) [76].

It is important to note that organs cannot develop and function normally without proper nutrients. During fetal development, a constant supply of energy, vitamins, minerals, and proteins is necessary for proper growth. It was shown that a lack of vitamins D and C leads to functional and structural disorders of the lungs [77,78]. On the other hand, a good diet with high doses of vitamins can help prevent serious lung illnesses. Specifically, in areas facing chronic vitamin A deficiency, maternal vitamin A supplementation before, during, and after pregnancy has been pivotal for lung maturation in offspring 9 to 13 years later. This suggests that early vitamin A interventions in communities with prevalent undernutrition can have enduring impacts on lung health [71]. Furthermore, vitamin A supplementation may also reduce the risk of chronic lung disease and sepsis in extremely low-birth-weight infants [79]. Therefore, a lack of nutrients during fetal development can lead to cellular and extracellular dysfunction, such as disruption of protein and DNA synthesis in the lungs, which can negatively affect lung growth and development [78,80,81].

Hence, the process of fetal lung maturation is complex and involves the interaction of various factors such as maternal, genetic, hormonal, and circulatory factors. Any disruption to this process can lead to negative outcomes. Understanding these factors can help develop personalized clinical interventions and improve maternal and neonatal health outcomes. Therefore, it is essential to conduct further research on fetal lung development to enhance clinical knowledge and guide the development of new therapeutic approaches.

### 2.3. Importance of Proper Fetal Lung Development for Postnatal Health

The postnatal stage is a challenging period in a person’s life, which involves a specific restructuring of the body’s organs, including the respiratory system. This stage is crucial for the body to adapt to new conditions. Unless there are genetic, hormonal, or other underlying issues, the body can cope with the stress of postnatal development without any difficulties. According to Zeltner et al., postnatal human lung development has two overlapping stages: the alveolar stage up to about two years, and the stage of microvascular maturation from the first months of birth to the age of 2–3 years [82]. Therefore, the proper development of fetal lungs is vital not only during the prenatal period but also for postnatal health and lifelong respiratory wellness. The intricate choreography of fetal lung development serves two central purposes: providing immediate respiratory function to the newborn and acting as a determinant for health trajectories across life [83]. In order to achieve these purposes, the next three significant aspects must be addressed, each of which is crucial in its own right.

The first, immediate respiratory function, is survival in the immediate postnatal period. With the transition from placental to pulmonary respiration after birth, the newborn’s lungs must be prepared for sudden exposure to air. The efficiency of gas exchange and the normal functioning of the respiratory system depend heavily on the developmental maturity of the lungs [84]. The occurrence of conditions such as neonatal respiratory distress syndrome, prevalent in premature infants with underdeveloped lungs, reflects the delicate balance and vital importance of fetal lung maturation [21,83].

The second, fetal lung development, not only paves the way for future respiratory health but also has implications for the individual’s long-term well-being [13,17,18]. This crucial period acts as a protective shield, molding the resilience of individuals against respiratory diseases such as asthma, chronic obstructive pulmonary disease (COPD, a progressive lung disease characterized by airflow limitation that is not fully reversible), and interstitial lung diseases. Evidence from various studies suggests that disruptions in fetal lung development can elevate the risk of developing these respiratory conditions and systemic health problems in adulthood [24,25,85,86,87]. Therefore, the proper orchestration of lung development pathways plays a pivotal role in preventing or establishing resilience against such diseases [83]. This concept aligns seamlessly with the broader theory of “Developmental Origins of Health and Disease”, emphasizing the profound impact of early-life conditions on future health trajectories [87,88,89].

Therefore, fetal lung development is a crucial point that shapes health throughout life. It is not just another step in the growth process. To develop effective preventive measures and therapeutic interventions, it is pivotal to understand the intricacies of this process. Genetic, hormonal, environmental, and nutritional factors all play a role in the course of fetal lung development. Specific disorders related to fetal lung development and their potential associations with adult diseases can generate valuable insights for future research and clinical practice. For example, Whitsett et al. suggest that understanding the genetic basis of rare lung diseases could help elucidate the molecular mechanisms controlling lung development [11]. Therefore, a multidisciplinary approach is necessary to comprehensively understand fetal lung development and its influence on lifelong respiratory health in order to advance medical research and practice.

## 3. Fetal Lung Development Disorders

Fetal lung development is an intricate and highly orchestrated process that begins early in pregnancy and continues through infancy. This development pathway is crucial for the well-being of a newborn, as the lungs must be fully functional at birth. Any disruption in this developmental pathway can lead to various disorders, many of which have profound and lasting consequences [90]. Immediate diagnosis and intervention often become essential to prevent lifelong complications, as several common and significant fetal lung development disorders demonstrate.

### 3.1. Respiratory Distress Syndrome (RDS)

**Figure 2:**
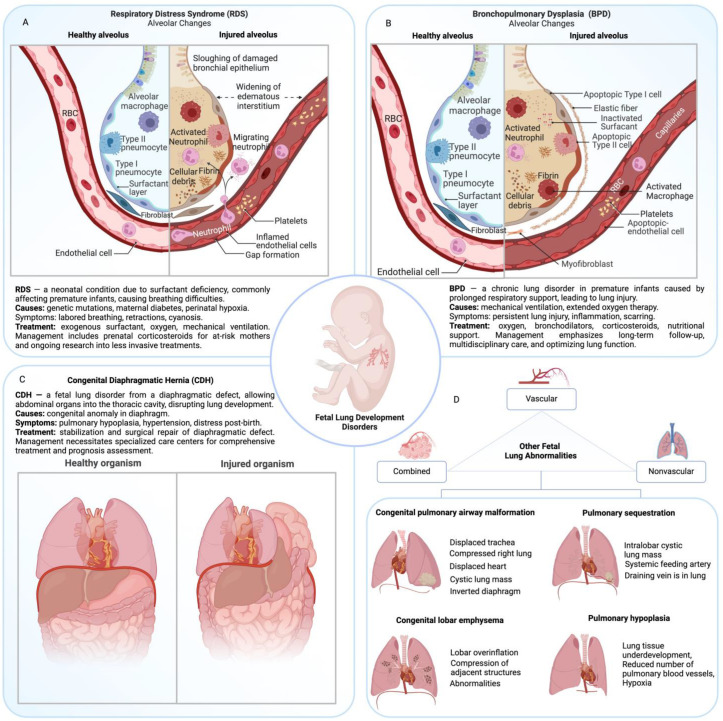
**Schematic illustration and brief description of main fetal lung development disorders.** (**A**) Respiratory distress syndrome (RDS), which is characterized by surfactant deficiency and labored breathing [91,92]. (**B**) Bronchopulmonary dysplasia (BPD), which is linked to premature birth and persistent lung injury [13,17]. (**C**) Congenital diaphragmatic hernia (CDH), where abdominal organs migrate into the thoracic cavity impeding pulmonary growth) [23,26], and (**D**) other fetal lung abnormalities including congenital pulmonary airway malformation (CPAM), Pulmonary Sequestration, Congenital Lobar Emphysema (CLE), and pulmonary hypoplasia (PH) [97,98,99,100].

Respiratory distress syndrome (RDS) is a respiratory condition primarily affecting premature infants, characterized by insufficient surfactant production, leading to difficulty in breathing and poor oxygenation shortly after birth (Figure 2A). Respiratory distress syndrome poses a significant challenge during fetal lung development. This condition, often associated with premature infants, is also referred to as hyaline membrane disease, owing to the protein-rich films that line the alveoli in affected infants [91,92]. A critical causative factor of RDS is the deficiency of surfactant, an essential substance that enables proper lung function by maintaining alveolar stability and facilitating efficient gas exchange [91,92]. It was shown that the deficiency or dysfunction in surfactant production can be caused by a variety of reasons, such as genetic disorders of the fetus (e.g., mutations like 4545C>G (p.Tyr1515*) in ABCA3 or low functional activity of NKX2.1), maternal diabetes, perinatal hypoxia, late preterm delivery, and ischemia, and delivery in the absence of labor [93,94,95,96].

Symptoms of RDS usually appear shortly after birth, evidenced by the newborn’s labored breathing, retractions (chest wall appears to cave in), and cyanosis (a bluish hue to the skin due to inadequate oxygenation) [91,92]. Treating RDS often entails administration of exogenous surfactant, supplemental oxygen, and, in severe cases, mechanical ventilation, providing respiratory support until the infant’s lungs can independently produce adequate surfactant [91,101]. In addition, supportive care, including thermoregulation, nutritional support, fluid and electrolyte management, and antibiotic therapy, remain important components of proper treatment [102,103].

Management of RDS has seen significant progress over time, propelled by advances in both prenatal and postnatal care. Mothers at risk of premature delivery receive corticosteroids to hasten fetal lung maturity, a practice based on landmark studies demonstrating a decrease in neonatal mortality and RDS incidence [40,41]. Furthermore, ongoing research continues to optimize therapeutic strategies, exploring avenues such as less invasive surfactant administration, reflecting the relentless pursuit of improving outcomes for infants affected by RDS [104,105].

### 3.2. Bronchopulmonary Dysplasia (BPD)

Bronchopulmonary dysplasia (BPD) is a notable chronic lung disease associated with premature birth (Figure 2B) [13,17]. This condition is frequently seen among infants who have undergone mechanical ventilation or prolonged oxygen therapy [106]. Unlike RDS where the core problem stems from surfactant deficiency, BPD arises due to persistent lung injury caused by extended respiratory support. Such interventions can elicit inflammation, scarring, and irreparable damage to the lungs [13,17,106].

Infants diagnosed with BPD often endure enduring respiratory complications that may extend into childhood or even adulthood. In a cohort study by McEvoy et al., a significant proportion of extremely low-birth-weight children with BPD showed reduced lung function at eight years of age compared to those without BPD [11,107]. Another longitudinal study by Gough et al. demonstrated an elevated risk of respiratory problems and reduced exercise capacity in young adults who had BPD during infancy [108].

Managing BPD typically necessitates a multifaceted approach, incorporating oxygen support, medications such as bronchodilators or corticosteroids, and nutritional support to promote lung growth and repair [109]. Acknowledging the challenges in BPD management, there is ongoing research aimed at optimizing mechanical ventilation strategies for preterm infants to avoid the development of BPD. This research focuses on fine-tuning the balance between providing sufficient respiratory support and minimizing lung injury, which is critical in the delicate lung environment of preterm infants. Due to its chronic nature and potential for long-term complications, BPD often mandates extended follow-up and multidisciplinary care, aiming to optimize lung function and holistic development of the child [109]. These efforts highlight the continuous evolution of treatment modalities and care practices, aiming to improve outcomes for infants afflicted with this condition. 

### 3.3. Congenital Diaphragmatic Hernia (CDH)

Congenital diaphragmatic hernia (CDH) is both a malformation and an etiological factor for lung disease, presenting a distinct array of complications within the spectrum of fetal lung development disorders. CDH is a congenital anomaly characterized by a defect in the diaphragm that allows abdominal organs to intrude into the thoracic cavity, subsequently impeding normal pulmonary development (Figure 2C) [23,26]. This intrusion can lead to pulmonary hypoplasia or underdeveloped lungs, and pulmonary hypertension or elevated blood pressure in the lung arteries, both of which may manifest as respiratory distress shortly after birth [110,111,112].

Early diagnosis of CDH is fundamental, and prenatal imaging techniques like ultrasound and MRI have improved detection rates [112,113]. These imaging methods not only allow for the identification of the condition but also the evaluation of the severity of lung hypoplasia and the presence of associated anomalies [112,113]. The presence of such anomalies requires timely treatment, and nowadays the focus of this therapy has been shifted towards innovative treatments like fetal endoscopic tracheal occlusion (FETO), a landmark procedure for severe CDH cases, which has shown promising results in improving lung growth and enhancing survival rates. However, the potential risks associated with FETO, including preterm birth and tracheal injury, demand further refinements in treatment strategies. Advances are being made in the development of a “self-unplugging” gel for tracheal occlusion that could eliminate the need for a secondary intervention, potentially reducing the risks associated with the current two-step protocol. This gel, possibly enriched with growth factors, is envisioned to foster alveolarization and support the normal differentiation of alveolar epithelial cells. By addressing lung hypoplasia and pulmonary hypertension head-on, these innovations aim to improve outcomes for infants affected by CDH. Additionally, future research is expected to introduce mechanisms that mimic fetal breathing, aiming to alleviate the harmful pressure gradients caused by tracheal occlusion and promote the expulsion of waste from the fetal lungs, marking a significant step forward in the treatment of CDH [114].

Postnatal management generally involves stabilization of the newborn’s condition followed by surgical repair of the diaphragmatic defect. However, the complexity and variability of CDH often necessitate a multidisciplinary approach at specialized care centers [113,115]. The severity of the disorder, associated anomalies, and the presence of pulmonary hypertension significantly affect prognosis, further underscoring the critical need for specialized, expert care in managing this condition [23,110].

### 3.4. Other Fetal Lung Abnormalities

Fetal lung abnormalities are a diverse group of disorders that encompass more than just the well-known conditions such as RDS, BPD, and CDH. There are a host of other conditions, each with distinct characteristics and unique management requirements (Figure 2D) [116].

One such example is congenital cystic adenomatoid malformation (CCAM), also known as congenital pulmonary airway malformation (CPAM) [97,117]. CCAM is a benign lung condition composed of cysts. It is commonly detected through prenatal ultrasound. While smaller lesions may not have a significant impact on the infant, larger ones can cause respiratory problems and may require surgical intervention.

Additionally, Pulmonary Sequestration presents another unique condition. This involves an area of non-functioning lung tissue that is disconnected from the usual bronchial tree and may receive blood supply from the systemic circulation. Such abnormal tissue often leads to recurrent respiratory infections and generally requires surgical resection [117,118].

Congenital Lobar Emphysema (CLE) is another lung development disorder, which is characterized by over-inflation of one or more lobes of the lungs. This results in compression of surrounding lung tissue and potential respiratory distress. In severe cases, surgical resection of the over-inflated lobe may be required [98,99].

These varied fetal lung abnormalities, among others, highlight the complex nature of disorders that can affect fetal lung development. They underscore the necessity for specialized, multidisciplinary care for infants affected by these conditions, as well as the continued need for research to refine diagnostic techniques and therapeutic strategies [98,99].

Thus, fetal lung development disorders present a diverse and complex array of conditions varying in severity, clinical presentation, and required treatments [116]. From the common RDS to the rarer conditions like CLE, the management of these disorders necessitates timely diagnosis, appropriate treatment, and often a multidisciplinary approach involving neonatologists, surgeons, and other specialists. Ongoing research endeavors continue to probe new diagnostic methods and therapies, enhancing our understanding of these challenging conditions [100,119]. The integration of prenatal diagnosis and innovations in neonatal care offers hope and reassurance to families navigating these difficult medical landscapes. Through continued research and collaboration among healthcare providers, the prognosis for infants affected by these disorders will likely continue to improve, reflecting the ever-evolving nature of medicine and medical technology. 

## 4. Linking Fetal Lung Development Disorders to Adult Diseases

**Figure 3:**
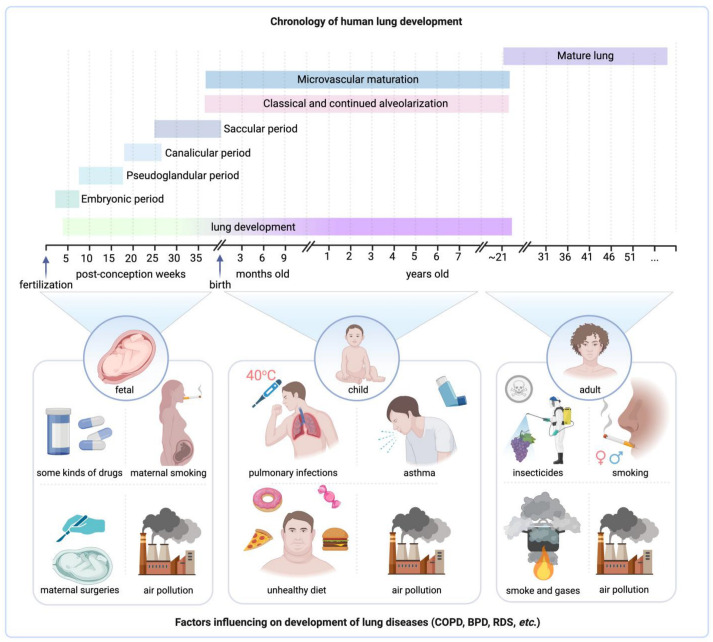
**Timeline of human lung development and main factors affecting this process.** The development of the lungs is a continuous process with overlapping stages, as most processes begin at the center and progress towards the periphery. The timing of microvascular maturation and the completion of alveolarization is unclear, resulting in fading bars. The embryonic period is not exclusively dedicated to lung development [21]. The risk factors for lung diseases are visually presented in a graph, categorized by the different stages of life: in utero and perinatal life, early childhood, and adulthood [120]. COPD—chronic obstructive pulmonary disease, BPD—bronchopulmonary dysplasia, RDS—respiratory distress syndrome.

The development of human lungs is a complex process that starts in early pregnancy and continues into adulthood, typically until around 20–25 years of age (Figure 3) [15,29]. This intricate timeline involves a plethora of events laying the foundation for a lifetime of respiratory function [30,42]. In recent years, scientific investigations have unraveled some of the complexities of this phenomenon, thereby deepening our comprehension of the machinery driving normal lung development as well as the elements that could lead to the emergence of disorders pertaining to this vital organ [29]. Stocks et al. summarized data showing that factors affecting fetal lung development can disrupt the normal timeline and have a lasting impact on an individual’s health [29]. Notably, some papers emphasize the link between chronic lung diseases, a primary source of morbidity and mortality worldwide, and irregularities in fetal lung development [15,29].

### 4.1. Long-Term Consequences of Fetal Lung Development Disorders 

**Figure 4:**
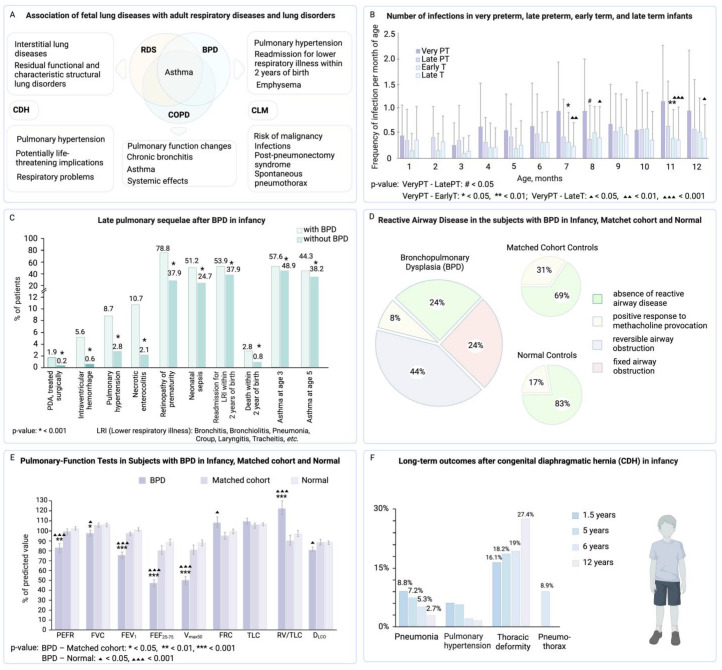
Overview of fetal lung diseases, their associations with adult respiratory conditions, and long-term health impacts on different infant groups. (**A**) Association of fetal lung diseases with adult respiratory diseases and lung disorders [18,121]. RDS—respiratory distress syndrome, BPD—bronchopulmonary dysplasia, COPD—chronic obstructive pulmonary disease, CDH—congenital diaphragmatic hernia, CLM—congenital lung malformations. (**B**) Number of infections in very preterm (VeryPT), late preterm (LatePT), early term (EarlyT), and late term (LateT) infants per month during their first year after birth (the study was carried out in Austria). Adopted from [123] © 2019 PLOS ONE. (**C**) Correlations between BPD in infancy and following long-term health disorders (the study was carried out in South Korea). PDA—patent ductus arteriosus, LRI—lower respiratory illness [126]. (**D**) Comparative analysis of reactive airway disease in infants with BPD, matched cohort controls (infants who were cared for in the intensive care nursery but did not receive mechanical ventilation, did not have BPD and major congenital anomalies, and lived nearby), and normal controls (healthy infants). The study shows that a large portion of individuals with BPD in infancy, specifically 52 percent, had reactive airway disease. This was significantly higher compared to the normal control group (the study was carried out in the USA) [107]. (**E**) Results of pulmonary function tests in subjects with BPD in infancy, matched cohort controls, and normal controls (the study was carried out in the USA). PEFR—peak expiratory flow rate, FVC—forced vital capacity. FEY_1_—forced expiratory volume in one second, FEF_25-75_—forced expiratory flow between 25 and 75 percent of vital capacity, V_max50_—maximal expiratory flow at 50 percent of vital capacity, FRC—functional residual capacity, TLC—total lung capacity, RV/TLC—the ratio of residual volume to total lung capacity, D_LCO_—diffusing capacity for carbon monoxide [107]. (**F**) Association between CDH in infancy and its long-term impact on children’s health (the study was carried out in Japan) [127].

The impacts of fetal lung development disorders are indeed profound and extend far beyond the neonatal period, affecting an individual’s lifelong health trajectory. These disorders can result in structural anomalies such as congenital diaphragmatic hernia, or functional abnormalities like surfactant deficiencies in respiratory distress syndrome, which can leave a lasting imprint on the respiratory system. Individuals who suffered from these disorders during infancy often experience chronic respiratory symptoms, reduced lung function, and are more prone to respiratory infections later in life (Figure 4A) [18,121]. This echoes the findings of a study by Pike et al., which indicated a long-lasting impact of neonatal lung disorders on adult health [121]. The association between early-life lung disorders and chronic respiratory conditions in later life has been extensively studied. For instance, Postma et al. emphasized that COPD is not only characteristic of the elderly but can manifest itself from birth (Figure 3) [120].

The long-term effects of these early disorders on lung function are varied but often substantial. A study by Ward et al. showed that children with a history of acute respiratory distress syndrome demonstrated reduced lung function, which persisted into adolescence [122]. Furthermore, these children had an increased susceptibility to respiratory infections, as premature birth itself compromises the immune system, resulting in increased vulnerability to infections, as supported by studies by Steiner et al. (Figure 4B) [123].

Beyond the direct impact on individuals, these disorders have substantial societal consequences as well. They necessitate specialized neonatal care, often involving extended hospital stays, use of intensive care services, and the need for various specialists. Moreover, long-term management of chronic respiratory issues places an ongoing burden on healthcare systems, families, and the affected individuals [124]. An economic analysis by Beam et al. estimated the medical costs of preterm birth (for the first 6 months of post-natal life) at over $8.4 billion (2016 USA) annually in the United States, illustrating the economic impact [125].

Thus, fetal lung development disorders are far from being solely a neonatal concern. Their effects reverberate throughout the life of the affected individual, impacting personal health, family life, and societal resources, underscoring the need for early intervention, ongoing research, and comprehensive long-term care strategies.

### 4.2. Association of RDS with Adult Respiratory Diseases 

Respiratory Distress Syndrome (RDS), commonly observed in premature infants, has been extensively studied due to its long-term implications. RDS arises from a deficiency of surfactant, a lipid–protein complex that plays a vital role in lung function, primarily in maintaining the patency of the air sacs within the lungs, or alveoli. When surfactant levels are inadequate, the alveoli collapse, resulting in severe respiratory distress, as indicated by a study by Ma et al. [128]. 

The advances in treatments for RDS, including exogenous surfactant therapy and mechanical ventilation, have substantially improved survival rates among affected infants [129]. In addition, surfactant administration has positive trends in infant survival with neurological impairment and reduction in respiratory morbidity during the first year of life [130,131]. However, the long-term respiratory prognosis can still be concerning (Figure 4A) [132]. Studies, including one by Shin et al., have found that children who were treated for RDS as infants may encounter a range of chronic respiratory issues, such as asthma, chronic obstructive pulmonary disease (COPD), and interstitial lung diseases (Table 1) [126]. Thus, the study showed that patients in the group with BPD were more likely to have asthma than in the group without BPD at the ages of 3 years and 5 years. Moreover, studies [133,134] have shown that respiratory illnesses in infants can have prolonged effects that persist into adulthood. Furthermore, additional research has indicated that residual functional and structural pulmonary disorders can be observed in adults who have previously had bronchopulmonary dysplasia [135,136].

It was reported that these chronic respiratory issues are more prevalent in adults with a history of RDS, emphasizing the need for ongoing monitoring, personalized care, and lifestyle modifications to manage these conditions effectively. As Cousins et al. pointed out, a history of RDS during infancy can serve as an important marker for potential respiratory health challenges throughout life [137].

The association between RDS and long-term respiratory complications also highlights the critical role of prenatal care and preventive strategies, aimed at reducing premature births and optimizing neonatal outcomes. A comprehensive review by McGoldrick et al. supports this view, advocating for maternal steroid administration to enhance fetal lung maturation when preterm delivery is expected [41]. Such interventions have been widely recognized and endorsed, leading to a significant decrease in the incidence of RDS and notably improving the long-term health of premature infants.

Therefore, while significant advancements have been made in managing RDS in the short term, the long-term respiratory outcomes can be challenging, necessitating a life-course approach to care for these individuals. Prevention strategies targeting premature births and early identification and intervention in neonates at risk of RDS are vital components of this approach.

### 4.3. Impact of BPD on Adult Lung Function and Respiratory Health

While BPD predominantly affects newborns, its repercussions can extend into adulthood, significantly impacting pulmonary health. The study conducted by Northway et al. underscores this point, revealing that young adults with a history of BPD in infancy exhibit notable reductions in lung function in comparison to those without BPD (Figure 4D,E) [107]. The study showed that 68% of subjects with a history of BPD in infancy had airway obstruction, 44% faced reversible airway destruction, and 24% had fixed airway obstruction. Hyperinflation was more common in these subjects than in controls and 24% of subjects had severe pulmonary dysfunction or current respiratory symptoms (Figure 4D,E) [107]. Similarly, BPD survivors had a lower exercise capacity and higher susceptibility to respiratory infections, validating the notion that BPD can limit physical capability and overall quality of life in adulthood [108,138,139]. Moreover, studies led by Wong et al. have shown that young people with a history of moderate to severe BPD may have lung dysfunction (functional and structural lung dysfunction), and in particular emphysema [133].

In response to these challenges, BPD has become a central subject of ongoing investigations within both neonatology and adult pulmonary medicine. Specialists in the field are diligently working to decode the complex pathophysiological underpinnings of BPD, aiming to devise preventive measures and therapeutic interventions. Recent studies, for instance, are focusing on the role of growth factors, cytokines, and additional elements in the development of BPD, exploring the potential of cell therapy as a means to therapeutically modify these components [140,141,142,143]. Additionally, neonatal care practices are being refined to reduce lung injury and prevent BPD. Promising results are seen with gentle ventilation methods, optimal nutrition, and meticulous fluid management [144,145].

Thus, the connection between BPD and adult respiratory health highlights the importance of ongoing monitoring and follow-up for BPD survivors. Early detection of lung function decline can facilitate timely intervention and disease management. By further investigating the causes and effects of BPD, healthcare providers can work toward reducing the long-term consequences of this disorder that occurs in early life.

### 4.4. Relationship between CDH and Adult Respiratory Issues

The intricate relationship between congenital diaphragmatic hernia (CDH) and long-term respiratory health provides valuable insight into how early developmental abnormalities can impact lifetime wellness and can cause lasting respiratory issues (Figure 4F) [146,147]. Although, modern treatments for CDH, including surgery, the use of nitric oxide (NO), extracorporeal membrane oxygenation (ECMO), and high-frequency oxygenation (HFO), can significantly reduce mortality associated with pulmonary hypertension and ventilator effects [148]. CDH patients are at an elevated risk of developing severe complications like pulmonary hypertension—a condition marked by heightened blood pressure within the pulmonary arteries [149]. This prevalent complication among CDH survivors can lead to severe repercussions, including right ventricular dysfunction and potential heart failure, highlighting the grave and potentially fatal nature of CDH [149,150].

Therefore, recognizing the link between CDH and long-term respiratory challenges highlights the importance of comprehensive, life-long healthcare management for these individuals. CDH survivors require ongoing medical and surgical care, including long-term medication, respiratory support, and multiple surgeries; these needs persist from infancy through adulthood [151,152]. After hospital discharge, CDH survivors face issues like respiratory problems, reflux, growth failure, developmental delays, hearing loss, hernia recurrence, and orthopedic issues (Figure 4F) [127,153,154,155]. These complications stem from underlying lung issues and surgeries. Additionally, nutritional and surgical complications can lead to further developmental problems [156,157].

Thus, monitoring for chronic respiratory issues, early detection of pulmonary hypertension, and intervention strategies are crucial in the care of CDH survivors. Additionally, advancements in perinatal care, including gentle ventilation strategies and new surgical techniques, aim to improve immediate neonatal outcomes, and can potentially reduce the long-term respiratory burden in CDH survivors [148]. Hence, the ongoing study of CDH and its lifelong impact serves as a testament to the lasting reverberations of early developmental disorders, emphasizing the need for holistic and vigilant healthcare approaches.

## 5. Mechanisms Underlying Associations between Fetal Lung Development Disorders and Adult Respiratory Diseases

To understand the link between fetal lung development disorders and adult respiratory diseases, a thorough investigation of the mechanisms connecting these two life stages is needed. This requires a multifaceted approach which covers various fields, including developmental biology and epigenetics. Such an approach can provide valuable insights into the developmental origins of adult diseases, the impact of transgenerational epigenetic modifications, and the effect of intrauterine and early environmental factors on lung development and future health outcomes.

### 5.1. Epigenetic Modifications in Lung Development and Disease

Epigenetic modifications, the heritable changes in gene expression that do not involve alterations to the DNA sequence, play a critical role in lung development and the predisposition to respiratory diseases [75,158]. These changes are often prompted by environmental influences and can determine individual susceptibility to various pulmonary conditions [109,159,160].

**Figure 5:**
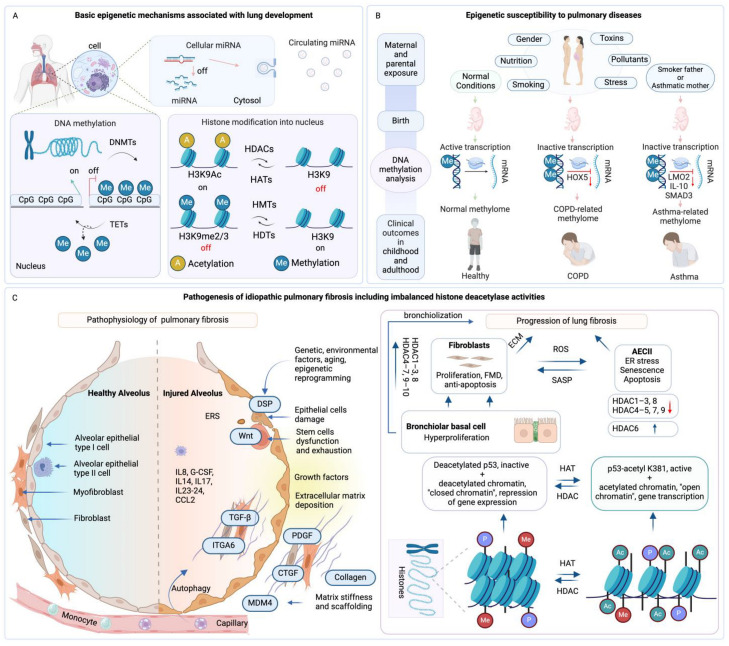
**Interplay of epigenetic mechanisms in lung development and disease susceptibility.** (**A**) Basic epigenetic mechanisms associated with lung development, illustrating cellular processes including DNA methylation, histone modification, and microRNA (miRNA) regulation within a cell. DNMT—DNA methyltransferase, HAT—histone acetyltransferase, HDAC—histone deacetyltransferase, HDT—histone demethyltransferase, HMT—histone methyltransferase, MV—microvesicle, TET—10–11 translocation methylcytosine dioxygenase [162]. (**B**) Epigenetic susceptibility to pulmonary diseases, highlighting the impact of gender, nutrition, environmental toxins, pollutants, stress, and parental smoking from birth through adulthood, alongside associated methylation patterns and transcription activities for normal, COPD, and asthma conditions. HOX5—hypermethylation of homeobox 5, LMO2—LIM domain only 2, IL-10—interleukin 10, SMAD3—mothers against decapentaplegic homologue 3 [162]. (**C**) Pathogenesis of idiopathic pulmonary fibrosis, delineating the roles of various factors in healthy and injured alveoli, the influence of imbalanced histone deacetylase activities on lung fibrosis progression, and the cellular and molecular responses involved in this process. ECM—extracellular matrix, HDAC—histone deacetylase, FMD—fibroblast-to-myofibroblast differentiation, ROS—reactive oxygen species, AECII—type-I/-II alveolar epithelial cell, SASP—senescence-associated secretory phenotype, HAT—histone acetyltransferase, Me—methylation, P—phosphorylation, Ac—acetylation [164,165].

The intricate process of lung development is regulated by a suite of epigenetic mechanisms [161,162]. DNA methylation, guided by DNA methyltransferases, acts as a molecular switch that can activate or silence genes, ensuring the proper progression from lung progenitor cells to a fully developed organ. Concurrently, histone modifications, which include the addition or removal of methyl and acetyl groups, remodel the chromatin landscape, thereby impacting gene accessibility and expression. This dynamic epigenetic environment is further nuanced by the activity of microRNAs (miRNAs) that modulate gene expression post-transcriptionally, fine-tuning the gene network essential for lung development (Figure 5A) [75,146,147,161,162,163].

From the earliest stages of life, epigenetic factors respond to environmental stimuli like maternal smoking, diet, air quality, and stress, sculpting the individual’s risk profile for respiratory diseases. Advances in epigenetic profiling have shown that specific DNA methylation patterns are associated with conditions such as chronic obstructive pulmonary disease (COPD) and asthma, potentially serving as early biomarkers for these ailments (Figure 5B) [162]. Specifically, idiopathic pulmonary fibrosis (IPF), a disease marked by progressive lung scarring, is linked to epigenetic alterations, particularly in histone deacetylase activities that govern fibroblast behavior and tissue repair processes, leading to the disease’s characteristic fibrotic remodeling (Figure 5C) [164,165,166].

**Figure 6:**
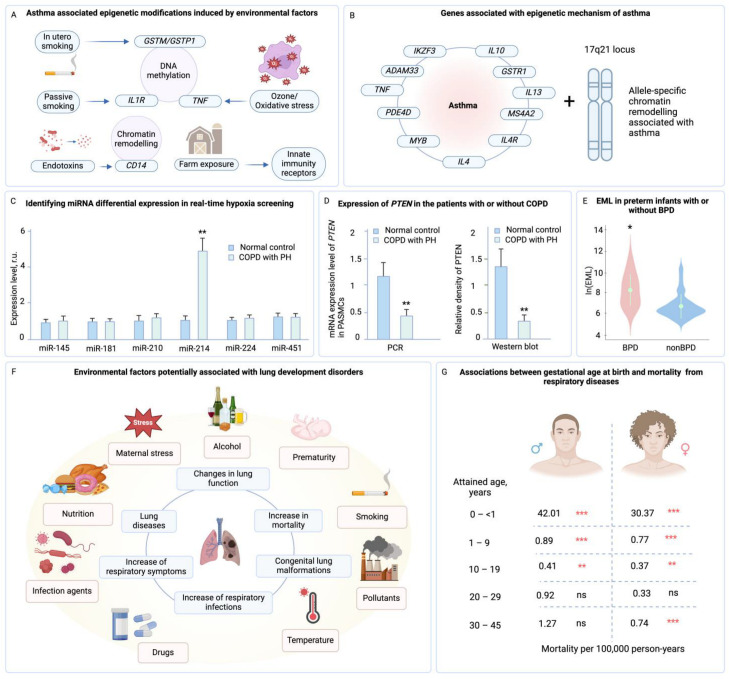
**Epigenetic and environmental determinants of lung development.** (**A**) Epigenetic modifications from environmental factors like smoking and ozone on gene expression related to asthma. GSTM1—glutathione S-transferase Mu 1, GSTP1—glutathione S-transferase P1, IL1R—interleukin-1 receptor, TNF—tumor necrosis factor [168]. (**B**) Key genes implicated in asthma through epigenetic changes [168]. (**C**) miRNA profiles in COPD patients with pulmonary hypertension (PH), suggesting their regulatory impact and diagnostic potential. Adopted from [169]. (**D**) Altered expression of the *PTEN* gene in COPD-associated PH, indicating epigenetic modulation. Adopted from [169]. (**E**) Comparison of lung changes in preterm infants with and without bronchopulmonary dysplasia (BPD). EML—epigenetic mutation load. Adopted from [170]. (**F**) Spectrum of environmental influences on lung development disorders [171,172,173,174,175,176,177,178,179,180,181,182]. (**G**) Correlation between gestational age at birth and respiratory disease mortality rates, highlighting epigenetic sensitivity during early development [183]. Asterisks indicate * *p* < 0.05, ** *p* < 0.01, *** *p* < 0.001, ns—non-significant: *p* > 0.05.

Extending beyond development, environmental factors continue to influence the epigenetic landscape into adulthood. Exposure to environmental toxins, such as tobacco smoke and pollutants, can lead to DNA methylation changes in specific genes, modulating the body’s oxidative stress response and impacting immune function, thereby increasing the risk of developing asthma (Figure 6A) [160,167]. Moreover, gene expression changes, including those in *ADAM33* and *IL10*, which have been linked to asthma through epigenetic mechanisms, suggest a complex interaction between genetic predisposition and epigenetic regulation (Figure 6B) [168].

Non-coding RNAs, such as microRNAs (miRNAs) have emerged as key regulators of gene expression with implications for pulmonary health. These molecules modulate gene expression post-transcriptionally, and studies have highlighted their substantial role in lung development and disease. For instance, research by Lui et al. has identified microRNA-214 (miR-214) as a regulator that targets the *PTEN* gene, which controls cell growth and death. The research showed that in COPD patients with pulmonary hypertension (PH), miR-214 was upregulated, resulting in decreased PTEN levels and increased cell proliferation in pulmonary artery smooth muscle cells. This indicates a role for miR-214 in the vascular changes seen in PH. The manipulation of miR-214 may thus offer new diagnostic and treatment strategies for PH in the context of COPD (Figure 6C,D) [169].

In preterm infants, epigenetic factors may also play a part in lung development disorders. Studies have shown that preterm infants with bronchopulmonary dysplasia exhibit distinct emphysema-like lung changes, suggesting that epigenetic modifications could have lasting effects on lung structure and function. A crucial finding supporting this notion is the analysis of the epigenetic mutation load (EML) in preterm infants, both with and without BPD. The study by Wang showed that EML was significantly higher in BPD samples than in those without BPD, indicating a substantial epigenetic impact on infants suffering from this condition (Figure 6E) [170]. This evidence highlights the importance of considering epigenetic factors in the context of lung development disorders and their potential to influence the prognosis of diseases like BPD. Moreover, these could potentially predispose to adult respiratory conditions such as asthma [168] or COPD [86,168,184].

To sum up, to prevent lasting consequences of development pathway disruption, immediate diagnosis, and interventions are important. As it has already been stated, despite any early interventions, permanent damages may include persistent reduced lung function or increased susceptibility to respiratory infections in later life. In addition, studies have revealed epigenetic mechanisms to be associated with birth and lung function trajectories from childhood to adulthood [185]. In this concept, the investigation of epigenetic biomarkers for early prediction of health outcomes may allow preventive and therapeutic interventions when necessary. 

### 5.2. The Impact of Environmental Factors during Intrauterine and Early Life Stages on Lung Development and Adult Health

The impact of environmental factors during intrauterine and early life stages on lung development and adult health underscores the intricate relationship between the intrauterine environment and early exposures, and their significant role in shaping fetal lung development with lasting effects on respiratory health. Factors such as birth weight, gestational age, nutrition, exposure to infections [160,174,186], environmental pollutants [66,167,187,188,189,190,191,192,193], and maternal stress are crucial in determining the development of the pulmonary system, potentially leading to congenital anomalies like CDH or other lung malformations. Additionally, the susceptibility of epigenetic regulation to environmental stimuli highlights the importance of evaluating genome-wide epigenetic changes (epigenomics) in diseases to discover new therapeutic targets and elucidate the pathophysiological mechanisms of diseases [161], further emphasizing the complex interconnections between environmental influences, genetic predisposition, and lung health outcomes (Figure 6F).

Hancox et al. have posited that disruptions in intrauterine growth could have enduring consequences for lung development [194], while Øistein Svanes et al. suggest that early-life adversity might sensitize the respiratory tract to the detrimental effects of cleaning products encountered later in life [195]. These findings underscore the critical period of lung maturation and the vulnerability of the developing respiratory system to external insults.

Prenatal exposures to harmful substances, such as smoke, alcohol, and certain medications, pose a risk to normal pulmonary maturation and are implicated in a spectrum of respiratory complications. The association of prenatal smoking with disrupted lung development, reduced pulmonary function from birth through adulthood, and increased susceptibility to respiratory infections and asthma, has been well-documented, along with concomitant epigenetic changes (Figure 6F) [171,172,173,174,175,176,177,178,179,180,181,182,196].

Moreover, postnatal factors including prematurity, infection, and the need for mechanical ventilation can persistently affect lung development and function. A significant study from Sweden highlighted the heightened mortality risk associated with preterm birth, attributing lung health as a primary contributor to this increased risk (Figure 6G) [183].

In addition, the relationship between fetal lung disorders and adult respiratory diseases involves a complex network of genetic and epigenetic interactions. Understanding these connections is vital for enhancing our predictive and preventive strategies for respiratory conditions [75,197]. The ‘developmental origins of adult disease’ hypothesis further illuminates how early life experiences imprint on health outcomes later in life. This concept, along with ongoing research into epigenetic modifications and their heritable impact, provides valuable insights into the mechanisms that influence lung development and function [75,147,162].

In summary, assessing the impact of intrauterine and early-life environmental factors on lung development is essential for advancing our comprehension of respiratory health. With an expanding base of knowledge, there is a growing opportunity to improve respiratory outcomes throughout an individual’s life. Interventions aimed at the earliest stages of development hold the promise of reshaping the trajectory of respiratory health for future generations, epitomizing the proactive ethos of preventive healthcare.

## 6. Exploring Potential Therapeutic and Preventive Strategies in the Context of Fetal Lung Development Disorders and Adult Respiratory Health

**Figure 7:**
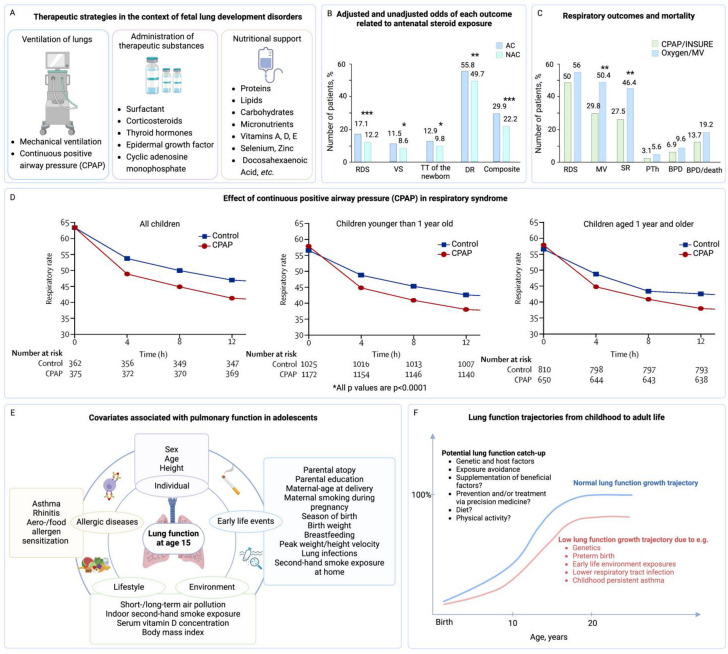
**Strategies and outcomes in fetal lung development interventions.** (**A**) Therapeutic strategies for managing fetal lung development disorders, including mechanical ventilation and CPAP, alongside the administration of surfactants and corticosteroids to enhance lung maturity and function [40,41]. (**B**) The impact of antenatal corticosteroid exposure on neonatal outcomes such as RDS, showing adjusted odds and beneficial effects of the intervention [198]. AC—antenatal corticosteroids, NAC—no antenatal corticosteroids, RSD—respiratory distress syndrome, vs.—ventilatory support, TT—transient tachypnea, DR—resuscitation in the delivery room, Composite—respiratory distress syndrome, transient tachypnea of the newborn, ventilatory support. (**C**) Various respiratory support outcomes, including the use of CPAP/INSURE and mechanical ventilation, on respiratory distress in newborns [199]. CPAP/INSURE—a bubble CPAP system, Oxygen/MV—oxygen via nasal cannula, RSD—respiratory distress syndrome, MV—mechanical ventilation, SR—surfactant requirement, Pth—pneumothorax, BPD—bronchopulmonary dysplasia. (**D**) The effectiveness of CPAP in managing respiratory syndrome across different age groups, demonstrating a significant reduction in respiratory rates compared to control groups. Adopted from [200] © 2017 Elsevier Ltd. (**E**) Covariates associated with pulmonary function in adolescents, highlighting the influence of environmental and lifestyle factors, as well as allergic diseases on lung function at age 15. Adopted from [201]. (**F**) Lung function trajectories from childhood to adult life, depicting potential catch-up in lung function and factors influencing normal and low growth trajectories, emphasizing the importance of early-life interventions in respiratory health. Adopted from [202] © 2017 F1000Research. Asterisks indicate * *p* < 0.05, ** *p* < 0.01, *** *p* < 0.001.

Early interventions play a crucial role in fetal lung development and have long-term implications for adult respiratory health (Figure 7A). To effectively manage fetal lung development disorders, a strategic and comprehensive approach is necessary that involves the expertise of obstetricians, neonatologists, and pediatric pulmonologists.

A significant area of early intervention is the administration of antenatal corticosteroids [40,41]. When administered to expectant mothers at risk of preterm birth, these corticosteroids expedite the maturation of the fetal lungs and stimulate surfactant production—a substance vital for proper lung function. This strategy has been shown in a study by Gyamfi-Bannerman et al., which exhibited a reduction in neonatal RDS and mortality in preterm infants following maternal corticosteroid administration (Figure 7B) [198]. Beyond immediate neonatal outcomes, the administration of antenatal corticosteroids has been shown to confer improved respiratory outcomes and a reduced incidence of severe complications such as intraventricular hemorrhage and necrotizing enterocolitis, solidifying their role in prenatal care [41,203,204].

In particular, Devender et al. demonstrated that treatment with antenatal corticosteroids significantly reduced the most serious adverse outcomes associated with prematurity compared to placebo and no treatment: perinatal death, neonatal death, RDS, moderate/severe RDS, the need for mechanical ventilation [41]. Another study revealed that, in infants born between 23 and 25 weeks of gestation, the administration of antenatal corticosteroids, as opposed to their non-use, was linked to a decreased incidence of death or neurodevelopmental impairment at 18–22 months [203]. Moreover, Mori et al. also showed that antenatal exposure to corticosteroids reduced the incidence of respiratory distress syndrome, severe intraventricular hemorrhage, and death in infants born at 24 to 29 weeks’ gestation. Thus, treatment with corticosteroids has been shown to be relevant in cases of preterm birth at 22–23 weeks of gestation [204].

The introduction of exogenous surfactant replacement therapy has been a pivotal advancement in neonatal medicine. Landmark trials from the 1980s established its efficacy in decreasing mortality and respiratory morbidities among preterm infants, underscoring the profound impact of early surfactant administration on respiratory function and the reduction in mechanical ventilation needs [205]. Administered early, exogenous surfactants can profoundly enhance respiratory function, diminish the requirement for mechanical ventilation, and curtail the risk of subsequent complications [101,104].

An additional pillar in the early intervention strategy can be respiratory support modalities, including continuous positive airway pressure (CPAP) and mechanical ventilation (Figure 7A), which are integral to the management of premature infants with respiratory complications [200]. The study by Tapia et al. demonstrated that early CPAP application could significantly reduce the need for subsequent mechanical ventilation, highlighting the importance of individualized respiratory strategies and careful monitoring to foster optimal lung development and minimize injury (Figure 7C,D) [199,200].

Postnatal nutritional support represents another key aspect of early intervention (Figure 7A) [68,80,81,144,206,207,208]. Ensuring adequate nutrition, especially for premature infants with fetal lung development disorders, is paramount for their overall growth and lung development. The role of nutrition has been affirmed by a wealth of evidence demonstrating that appropriate caloric intake and sufficient nutrients, including essential vitamins (A, C, D, etc.) and minerals, have a direct influence on lung growth, development, and repair after injury [209,210].

Unraveling the correlation between fetal lung development disorders and adult diseases provides a valuable window into preventive strategies and management of respiratory issues later in life. The incorporation of this knowledge into clinical practice facilitates the implementation of targeted preventive measures and interventions, with the potential to lighten the load of respiratory diseases in adulthood.

In the context of prevention and management, continuous monitoring and early detection of respiratory problems stand out as key strategies [211,212]. Individuals with a history of fetal lung development disorders warrant ongoing medical surveillance across their lifespan. Evidence from studies like the Danish National Birth Cohort emphasizes the importance of early detection in mitigating the severity of conditions like asthma and COPD, enabling timely intervention and better disease management [213].

The adoption of lifestyle modifications plays an instrumental role in reducing the risk of respiratory diseases. Advocacy for healthy choices, such as abstinence from smoking and minimization of exposure to environmental pollutants, can be significantly beneficial, especially for individuals predisposed due to fetal lung development disorders [66,186,189,190,214]. In a study by Gudmundsdottir et al., it was shown that the respiratory function (t_PTEF_/t_E_) of newborns up to 3 months depends on the lifestyle of the pregnant mother [214]. This approach aligns with a plethora of evidence indicating that lifestyle factors can modulate respiratory health trajectories, emphasizing the need for health promotion and disease prevention strategies that encourage healthy behaviors (Figure 7E,F) [188,201,202].

Respiratory rehabilitation programs cater to adults with a history of fetal lung development disorders. These initiatives aim to ameliorate lung function, boost exercise capacity, and enhance overall respiratory health through targeted exercises and respiratory therapies [215,216,217,218]. Studies, like the one conducted by Román et al., reinforce the value of pulmonary rehabilitation in improving respiratory symptoms, exercise tolerance, and quality of life in patients with chronic respiratory diseases [216].

Personalized care is a cornerstone in the effective management of individuals with a history of fetal lung development disorders. Healthcare plans, uniquely tailored to accommodate an individual’s medical history, current respiratory function, and potential risk factors, can bolster disease prevention and improve treatment outcomes [215,219,220]. The utility of personalized care is increasingly recognized in modern medicine, with numerous studies emphasizing its potential to revolutionize the management of chronic diseases like asthma and COPD [221,222,223,224].

Therefore, early interventions and preventive strategies can leave an indelible impact on the quality of life and long-term health outcomes for those affected by fetal lung development disorders. By acknowledging the developmental origins of health and disease, healthcare professionals can more effectively navigate the intricate interplay between fetal lung development and adult respiratory health. This comprehensive approach not only improves individual well-being but also contributes to reducing the burden of respiratory diseases at a population level.

## 7. Exploring Future Research Directions in the Domain of Fetal Lung Development Disorders and Associated Adult Diseases

The medical and scientific community has made significant strides in comprehending the association between fetal lung development disorders and adult diseases. However, despite these advancements, a multitude of areas still demands further exploration to enrich our understanding and enhance clinical outcomes. These future research endeavors should target specific areas of interest to generate insights and solutions that are currently elusive.

Longitudinal follow-up studies are instrumental in grasping the trajectory of respiratory health over time, particularly for individuals with a history of fetal lung development disorders. These long-term studies offer a wealth of information that aids in identifying risk factors, discerning disease progression patterns, and determining the impact of early interventions on adult respiratory outcomes. An example of this can be found in the seminal Avon Longitudinal Study of Parents and Children (ALSPAC), which monitors the health of individuals from birth to adulthood, generating invaluable data on the long-term outcomes of early-life events [191,225]. 

An illustrative instance of this can be seen in the pioneering Avon longitudinal study of parents and children (ALSPAC), which tracks health trajectories from birth to adulthood, offering invaluable insights into the enduring impacts of early-life experiences [200,226]. For example, in the study conducted by Hansell et al., it was demonstrated that exposure to local and distant road traffic particulate matter with a diameter of ≤10 μm during the first trimester could lead to a modest yet significant reduction in lung function at eight years of age. The study revealed that at eight years, exposure to road traffic during the initial trimester was correlated with lower FEV1 and FVC. Comparable relationships were observed for exposures during the second and third trimesters, as well as during the first six months, the subsequent six months, and up to seven years of life. These associations were more pronounced in boys, children whose mothers had lower levels of education, or who smoked during pregnancy. Particulate matter 10 from all sources during the third trimester was significantly linked with lower FVC z-scores. However, no substantial negative associations were noted at age 15 years [191]. 

In another study within the ALSPAC framework, it was also shown that by utilizing repeated data on greenspace and lung function at ages eight, 15, and 24, the presence of greenness within a 100 m buffer was associated with higher FEV1 and FVC, with increases of 11.4 mL and 12.2 mL, respectively, per interquartile range increase. Similarly, the presence of urban green spaces within a 300 m buffer correlated with higher lung function (20.3 mL for FEV1 and 23.1 mL for FVC, respectively). Notably, these associations remained independent of factors such as air pollution, urbanicity, and socio-economic status. Furthermore, the lifetime average greenness within a 100 m buffer and the proportion of agricultural land within a 300 m buffer were linked to improved lung function at the age of 24 [225].

**Figure 8:**
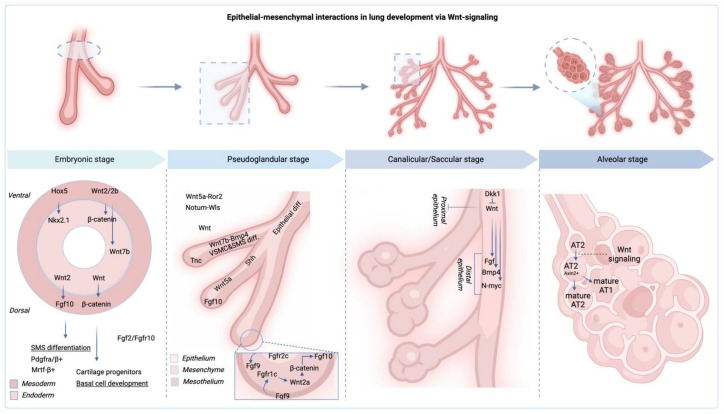
**Stages of lung development influenced by Wnt signaling.** Embryonic stage—HOX5 in the mesenchyme directs Wnt2/2b in the endoderm to form lung progenitors and initiate smooth muscle and cartilage development. Pseudoglandular stage—lung branching morphogenesis is driven by mesenchymal Wnt5a and regulated by Notum, with Wnt7b-BMP4 axis aiding in tissue growth. Canalicular/Saccular stages—Wnt signaling, modulated by DKK1, differentiates the lung epithelium, while high Wnt levels promote distal airway formation. Alveolar stage—Wnt-responsive ATII cells guide the maturation of alveolar structures. Adapted from [229].

Mechanistic studies are paramount in shedding light on the intricate molecular pathways such as Wnt signaling pathway and epigenetic mechanisms that tether fetal lung development disorders to adult respiratory diseases. Investigating gene expression patterns, the role of specific proteins, signaling pathways, and epigenetic modifications can furnish insights into potential therapeutic targets [166,227,228,229,230,231]. For example, Aros et al. illuminates the role of the Wnt signaling pathway in lung development, demonstrating the importance of such mechanistic research. The prominence of the Wnt signaling pathway in these developmental processes, as highlighted in Figure 8, showcases the critical regulatory role it plays from the embryonic through to the alveolar stages. This pathway’s involvement in cellular differentiation, growth, and organization within the lung substantiates its significance as a focal point in understanding pulmonary pathophysiology. By comprehending the nuances of Wnt signaling, researchers can potentially unravel the molecular underpinnings of lung development disorders, paving the way for novel interventions in managing respiratory ailments that manifest across the lifespan [229].

Emerging research also indicates that the inter and transgenerational effects of fetal lung development disorders may be more significant than previously assumed [232]. Investigations into whether adverse lung development in one generation affects respiratory health in subsequent generations can broaden our understanding of the wider impact of early-life events. Thus, in particular, research of Gregory et al. showed that in mice distinct traces of methylation, were identified in dendritic cells in the F1, F2, and F3 generations if the organism was exposed to environmental particles (diesel exhaust particles (DEP) and (concentrated urban air particles) CAPs) during pregnancy, and also observed the changes in the EOS’s levels (in vivo). These findings indicate that even a single exposure to DEP and VP during pregnancy can facilitate transgenerational maternal transmission of an increased asthma risk to F1, F2, and to a lesser extent, F3 generations [226]. The study by Pembrey et al. summarizes the data on transgenerational responses to environmental challenges and highlights the potential impact of such influences [233].

The role of intrauterine environmental factors, including maternal exposures and placental function, in determining fetal lung development and the ensuing risk of adult respiratory diseases is another aspect requiring deeper research. For example, the association between maternal during pregnancy and impaired lung function in offspring is well-documented, but the extent to which these effects persist into adulthood needs further exploration [174,176,182].

Moreover, the development of personalized medicine approaches can considerably enhance disease prediction accuracy and improve tailored therapeutic strategies [223]. Considerations should include an individual’s genetic makeup, epigenetic profile, and early-life exposures. Personalized medicine is increasingly recognized as the future of healthcare, with a growing body of the literature indicating that personalized therapeutic approaches, such as pharmacogenomics-based treatments, can significantly improve patient outcomes [221,234].

Parallel to these research directions, identifying potential therapeutic targets is crucial for devising effective interventions to improve respiratory outcomes in individuals with a history of fetal lung development disorders.

Surfactant enhancement, for instance, either by boosting natural surfactant production or by improving the formulation of exogenous surfactants, could improve lung function in neonates and adults struggling with RDS or other surfactant-related issues [132]. This approach is supported by extensive research into surfactant proteins and their role in lung health and disease.

Anti-inflammatory therapies targeting inflammation and oxidative stress pathways may be promising in mitigating long-term lung injury and reducing the risk of respiratory diseases associated with fetal lung disorders like BPD [235,236,237]. For instance, in the study conducted by Chen et al., in vivo findings highlighted the dual effects of ibuprofen on lung development. Specifically, while ibuprofen negatively impacted angiogenesis, it demonstrated positive effects on alveolarization and reduced inflammation. As a result, the study concluded that while ibuprofen shows promise in certain aspects of lung development, its application in treating premature infants with BPD must be approached with great caution due to its mixed effects. This underscores the complexity of drug effects on developing tissues and the need for careful consideration in their clinical use [235]. In another study conducted in vitro, the focus was on exploring the therapeutic potential of budesonide, a glucocorticoid, in addressing the critical issue of lung inflammation that leads to BPD in premature infants. Unlike traditional treatments that rely on systemic administration of drugs like dexamethasone, which are associated with notable adverse effects, this research highlighted the advantages of using budesonide when delivered directly into the lungs via surfactant. The in vitro study meticulously cultured second-trimester fetal lung explants with budesonide, employing a range of sophisticated methodologies including microscopy, immunoassays, RNA sequencing, and liquid chromatography to assess its efficacy. The findings were significant, showing that budesonide rapidly reduced levels of pro-inflammatory chemokines such as IL-8 and CCL2 within just a few hours, with a pronounced effect observed at the 12 h mark. This reduction was not only swift but also potent, with budesonide demonstrating a substantially higher effectiveness compared to dexamethasone, all while maintaining stability and avoiding the adverse effects typical of systemic therapies. These in vitro results underscore budesonide’s potential as a safer, more targeted approach to preventing or treating BPD in neonates, marking a promising direction for future clinical applications [236].

These studies have underscored the pivotal role of inflammation in the development of chronic lung diseases and pointed to the potential therapeutic efficacy of anti-inflammatory agents.

Regenerative therapies, such as stem cell-based approaches, are another prospective area for therapeutic development. These novel treatments could repair lung tissue damage incurred by fetal lung development disorders and promote lung regeneration, a concept backed by emerging preclinical and clinical studies demonstrating the potential benefits of stem cell therapies for lung diseases [238,239,240].

Epigenetic modulators offer another direction for potential therapeutic intervention [241]. Understanding the epigenetic modifications influencing gene expression during fetal lung development could usher in the development of epigenetic drugs for adult respiratory diseases. Epigenetic changes, such as DNA methylation and histone modification, are increasingly recognized as key factors in disease development and progression, leading to the emergence of epigenetic therapies [147,241].

Lastly, implementing preventive interventions such as maternal lifestyle modifications and targeted neonatal care may help reduce the incidence and severity of fetal lung development disorders and their associated adult respiratory consequences. Studies consistently highlight the significant impact of preventive measures on health outcomes, affirming the adage that prevention is indeed better than cure [88,201].

As research in this domain advances, new insights into the complex relationship between fetal lung development disorders and adult diseases will continue to surface. By channeling research efforts towards these future directions and potential therapeutic targets, we can stimulate the development of innovative interventions and strategies aimed at improving respiratory health and overall well-being for individuals affected by fetal lung disorders across their lifespan. The promise held by these research pursuits extends beyond individual health, with the potential to make considerable contributions to public health and foster a future where respiratory diseases are more comprehensively understood, preventable, and effectively managed.

## 8. Conclusions and Future Perspectives

In this comprehensive review, we explored the intricate relationship between fetal lung development disorders and the onset of adult diseases, offering a detailed analysis of the developmental origins of health and disease. The journey from the embryonic phase to the alveolar stage in fetal lung development is a complex ballet of biological processes, pivotal for postnatal respiratory function [9,58]. This journey, influenced by genetics, hormonal inputs, and maternal and fetal circulatory impacts, is crucial for the lung’s maturation. Disruptions in this process, evidenced in conditions like RDS, BPD, and CDH, have far-reaching implications for respiratory health across the lifespan.

Our exploration emphasizes the enduring impact of early-life events on adult health outcomes, advocating for a life-course approach to healthcare. This approach, focusing on early interventions and preventive measures, can significantly improve neonatal outcomes and mitigate long-term respiratory health issues. Public health policies and healthcare planning will benefit from this understanding, especially in reducing the burden of adult respiratory diseases. The review also highlights the need for further research into the mechanisms of fetal lung development and potential transgenerational effects. Such research could unveil new therapeutic targets and pave the way for personalized medicine in preventing and managing adult respiratory diseases.

In conclusion, the connection between fetal lung development disorders and adult diseases is a critical area of study with significant implications for public health and medical practice. Leveraging this knowledge can lead to a future where respiratory diseases are better understood, preventable, and effectively managed, promising improved health and well-being globally.

## Data Availability

The raw data supporting the conclusions of this article will be made available by the authors on request.
